# Complex congenital lung malformation resection by uniportal VATS—case report

**DOI:** 10.1093/jscr/rjab069

**Published:** 2021-04-13

**Authors:** Arpad Hasenauer, Céline Forster, Amaya Ojanguren, Michel Gonzalez

**Affiliations:** Service of Thoracic Surgery, University Hospital of Lausanne (CHUV), Lausanne, Switzerland; Service of Thoracic Surgery, University Hospital of Lausanne (CHUV), Lausanne, Switzerland; Service of Thoracic Surgery, University Hospital of Lausanne (CHUV), Lausanne, Switzerland; Service of Thoracic Surgery, University Hospital of Lausanne (CHUV), Lausanne, Switzerland

## Abstract

Bronchial atresia (BA) is a rare congenital pulmonary airway malformation. It is characterized by the focal stenosis of a proximal segmental bronchus associated with peripheral mucus impaction and hyperinflation of the obstructed lung segment. Most cases are identified during neonatal period or childhood. When diagnosed in adults, BA may present with recurrent infections, pneumothorax and destruction of affected parenchyma. Thoracoscopic approach to BA has proved challenging in adult patients because of repeated infections and subsequently, its inflammatory status. Herein we present a case of a 26-year-old female with left side recurrent pneumonia and pneumothorax past history. A chest computed tomography revealed a complex congenital bronchial atresia involving the left upper lobe and basal segments, associated to vascular anomalies. She underwent a successful uniportal VATS left upper lobectomy and resection of basal segments. Uniportal VATS approach is an effective and safe treatment for the management of complex congenital lung malformation.

## INTRODUCTION

Bronchial atresia (BA) pertains to the group of congenital pulmonary airway malformations. It is characterized by focal stenosis of a proximal segmental bronchus associated with peripheral mucus impaction and hyperinflation of the obstructed lung segment [1]. Most cases are clinically asymptomatic and are identified incidentally in patients examined for other clinical indications during neonatal period or childhood. When diagnosed in adults, BA may present with recurrent infections and pneumothorax. At the final stage, pulmonary parenchyma degradation may occur. In spite of its benign nature, surgery is indicated in symptomatic patients to avoid the previously described complications. However, the thoracoscopic approach to BA has proved challenging in the adult patient because of repeated infections and subsequently, its inflammatory status.

Herein, we describe the uniportal procedure for a complex BA comprising the left upper lobe plus basal segments.

## CASE REPORT

A 26-year-old female patient with left side recurrent pneumonia and pneumothorax past history was referred to thoracic surgery department after a chest computed tomography (CT) revealed a complex congenital pulmonary airway malformation in the context of chest pain.

**Figure 1:**
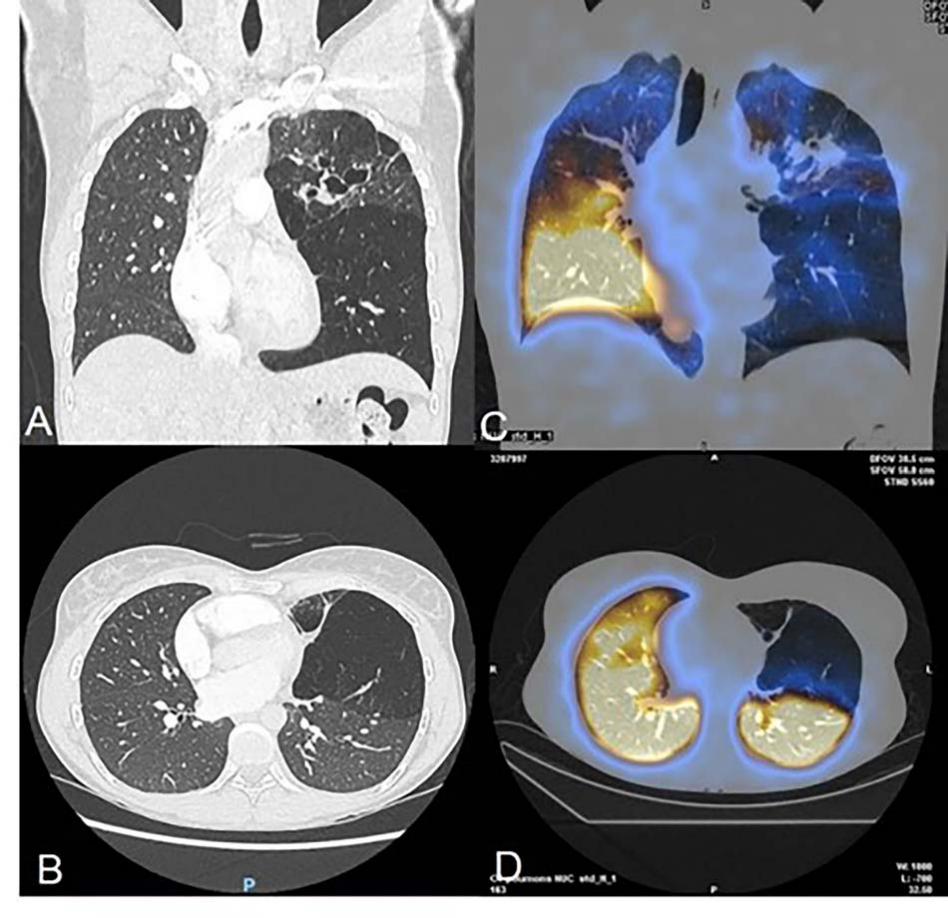
(**A–B**) Chest CT-scan showing the complex congenital malformation with destruction of the left superior lobe with bronchiectasis and a voluminous bulla of the left lower lobe. (**C–D**) Perfusion scintigraphy revealing no perfusion of the left upper lobe and bulla of the left lower.

More precisely, the contrast enhanced CT showed a partial stenosis of the upper division segmental bronchus of the left upper lobe, and an atresia of the anterior segmental and the lingular bronchus associated with distal bronchiectasis and superinfection signs (Fig. 1). The left lower lobe presented BA at the level of a supernumerary paracardiac bronchus and an anterobasal segmental bronchus associated with voluminous pulmonary bullae. Some anatomical vascular variations, such as common venous trunk, and a common arterial trunk for the anterior segment of the upper division and the lingula were identified. The right lung did not present anomalies. The ventilation perfusion scintigraphy showed left hypoperfusion corresponding to the left upper lobe (7% of total lung function). After multidisciplinary meeting surgical treatment was proposed.

General anesthesia was managed with a right double-lumen tube. The patient was positioned on right lateral side and the table was flexed (video 1). A 4-cm incision was performed in the fifth intercostal space in anterior axillary line. The surgeon and the assistant were positioned on the anterior side of the patient. A 10-mm 30° thoracoscope was used. A dissector, a hook monopolar cautery and an Ultracision Harmonic scalpel were used for dissection and coagulation, respectively. All vessels were divided by endoscopic staplers.

In this particular patient, firm pleuropulmonary adhesions were found. Dissection started by incising the mediastinal pleura around the hilum. Then, all arterial branches were carefully dissected on a sufficient length to avoid misidentification. First, A_1a_ was identified and cut by an endoscopic stapler. This step facilitated the exposure of A_1b_, that was divided as well. At this point, a common arterial trunk for A_3 + 4 + 5_ and a common left venous trunk were identified. Then, A_3 + 4 + 5_ and upper vein were isolated and cut. Dissection continued with the left upper bronchus, that was divided by an endostapler. Next, A_2_ was perfectly identified and cut. The specimen was removed in a retrieval endobag.

The operation proceeded in the lower lobe. First, the lung was ventilated. This maneuver facilitated to perform a demarcation line (between the bullous lung parenchyma and normal parenchyma) that was marked by electrocautery. At this point, we decided to conduct a parenchyma sparing technique by means of harmonic scalpel in order to preserve the maximal amount of healthy lung and reduce distortion caused by endostaplers. A fibrin sealant was applied on the depleurized parenchyma to reduce postoperative air leakage.

**Figure 2:**
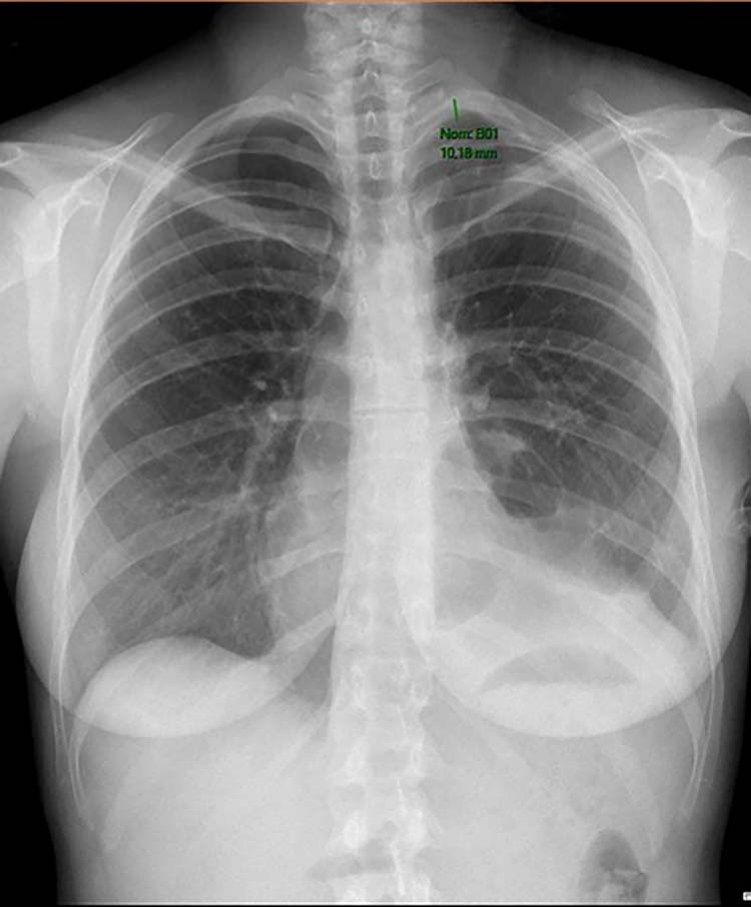
Chest radiography showing small residual pneumothorax after chest tube removal.

The overall surgical time was 159 min. The operation was uneventful. On the 10th postoperative day chest tube was removed (Fig. 2). No complications were noticed besides a prolonged air leak that was treated conservatively.

## DISCUSSION

Congenital BA is a rare disorder with an estimated prevalence of 1.2 cases per 100 000 population [2]. The cause of BA has not been elucidated but two main theories had been exposed. The first estates that during normal lung maturation, proliferating cells lose their connection with the developing bronchial bud. The second hypothesis is that a vascular insult to the bronchial bud during fetal development leads to obliteration of an already completed bronchus. As the distal parenchyma is normally developed, secreted mucus leads to distal mucoid impaction and collateral ventilated alveoli cause air-trapping. Such anatomic conditions promote repetitive infections, lung parenchyma destruction and pneumothorax [3]. Although the treatment of asymptomatic patients is a matter of controversy in adults, there is a well-accepted consensus on operating on symptomatic patients [3–7].

In recent years minimally invasive surgery has been recognized as safe for the treatment of BA [3, 8], however, the uniportal approach has not been reported. In our experience, the best operative strategy for BA should include a contrast enhanced CT-scan to evaluate first, the extension of the congenital anomaly and second, the eventual appearance of anomalous vessels. In addition, a bronchoscopy to identify the blind-ending segmental bronchus can be helpful for the diagnosis, even if it may not be visible if the occlusion is beyond bronchoscopy’s range.

On the basis of adult and symptomatic patients, the following specific considerations should be taken into account as follows. As BA is benign, it seems advisable to spare as much parenchyma to preserve pulmonary function, thus, sublobar resection needs to be encouraged. Also, a pleuropulmonary inflammatory condition should be expected. In this particular setting, if major infection is present with cicatricial tissue and large inflammatory lymph nodes surrounding pulmonary vessels, we encourage to perform a thoracotomy. In any case, minimally invasive approach should be the first approach. And finally, because of the infectious sequellae and damaged lung tissue, prolonged air leaks may be foreseen because of the loss of elasticity of the parenchyma that impairs pulmonary re-expansion.

Our video shows that uniportal approach for a complex case of congenital BA is feasible. Limited inflammatory status and vascular anatomical variations should not be a limiting factor for uniportal VATS resection if planned carefully.

From this report, uniportal VATS approach is considered to be an effective and safe treatment for the management of complex congenital BA.

## CONFLICT OF INTEREST STATEMENT

None declared.

## FUNDING

None.

## ETHICAL STATEMENT

The authors are accountable for all aspects of the work in ensuring that questions related to the accuracy or integrity of any part of the work are appropriately investigated and resolved.

## INFORMED CONSENT

Written informed consent was obtained from the patient for publication of this manuscript and any accompanying images.

We present this case in accordance with the CARE Guideline.

## Supplementary Material

Video_JSCR_Complex_congenital_lung_malformation_resection_by_uniportal_VATS_rjab069
